# War zone refugia? Establishing a baseline for protected waterbirds in a wildlife refuge flanked by agriculture and militarization

**DOI:** 10.1186/s13104-018-3334-5

**Published:** 2018-04-02

**Authors:** Michael H. Parsons, Prameek M. Kannan

**Affiliations:** 1 Department of Biological Sciences, Fordham University, Bronx, NY 10458 USA; 2 Department of Biology, Pace University, Pleasantville, NY 10570 USA

**Keywords:** Conservation monitoring, Avifauna and militarization, Remote wetland biodiversity, War zone refugia

## Abstract

**Objectives:**

The welfare of threatened fauna should not be assumed merely because their refuges have been designated with protected status. This is particularly true in geographical areas where social/military events drive an under-reported, but potentially lethal, type of human–wildlife interaction. Waterbirds of Gharana Wetland Conservation Reserve consist mostly of threatened species. However, as occurs globally, ‘protected’ fauna near contested borders are sometimes affected by military forces. As part of a larger project to document regional avifauna, we report the seasonal status of waterbirds in order to help establish a baseline for comparing conservation of wildlife within contested areas to that of fauna in more secure refuges. We examined 24 avifauna surveys for relationships between seasons, temperature, individuals and species.

**Results:**

28 of 61 waterbird species were rare. We found seasonal variations in individuals (*F*_3,731_ = 3.82; *P *< 0.01) and species (*F*_3,11_ = 5.81; *P* < 0.05) with a major influx in late winter, rather than autumn. Thus, while this sanctuary serves as an over-wintering site, it is also a stop-over site for high-altitude migrations. While providing this baseline, we offer a reminder that the welfare of wildlife in protected areas should be monitored seasonally, with the ongoing threats to their conservation, carefully documented.

## Introduction

Human militarization can influence the behaviors and population status of wildlife [1, 2] however, few studies documenting this type of human–wildlife conflict (HWC) focus on protected refuges or wildlife sanctuaries [3]. Indeed, gaining the formal protection of a wildlife sanctuary is often considered an end unto itself [4]. The Gharana Wetland Conservation Reserve (GWCR; 32°32′28″N; 74°41′27″E; 281 m asl) is a critical wetland (~ 0.75 km^2^) situated along the international Indo-Pakistan border. While wetlands provide shelter for more than 12% of all animal species and 40% of all birds [5], this wetland may be especially important because it is found along the Central Asian flyway (Palearctic–Oriental) for winter and summer migrations [6]. The relatively mild winters and abundant resources attract rare and threatened species from as far away as Siberia and Mongolia. However, as occurs in regions throughout the world—despite the international and local designations for protection [7, 8]—the local political and economic conditions challenge the “protected status” of endangered and threatened wildlife.

The principal threats to conservation in GWCR relate directly to its proximity to a contested geopolitical border. Following independence in 1947, both India and Pakistan claimed the same ‘line-of-control’ [9]. Thus, for the past 70 years, there has been varying levels of military activity, including use of 82 mm mortars, in the area [9]. These shells can influence the wildlife directly through ordinance-strikes, or indirectly through reverberation [10]. In 2003, a formal ceasefire was declared. Ironically, this action increased anthropogenic pressure and revealed a complex dynamic between militarization and agriculture, found here [10], and other regions around the world [11]. Whereas, the declaration of a ceasefire did not completely halt the shelling, but instead limited the threat enough so that farmers moved into cultivate the wetland for farming.

### Complex dynamics between militarization and agriculture

Locally, farmers not only endure stray firing, they also compete with wildlife for crops. As occurs in most avifauna refuges, migrant visitors are herbivores, and thus consume seeds, saplings, wheat and even crops such as basmati rice, a local mainstay. When migrants arrive in winter, palatable shoots of wheat and rice seedlings are already germinating [12], Consequently, waterbirds are not only threatened by shelling, but also by farmers who chase them with firecrackers which (ironically, if not purposefully) mimic gunfire and reverberation [13].

Additionally the refuge is compromised because villagers deposit their wastes into the wetland, thus, exacerbating silting [13]. Pollution from fertilizers and domestic animal excreta are further threats. Lastly, some farmers intentionally dump soil into the wetland to increase farmable surface area. The take home lesson is that there is a complex and under-reported dynamic between military and agricultural conflicts. Unfortunately, wildlife in such protected habitats may not be as sheltered as we suppose [14].

From a global perspective, decreases in the abundance or fluctuations of waterbirds are particularly important to document in areas such as these [15]. The conditions of which, may be exacerbated by human presence and ‘both types’ of disturbance. As such, the status of water-birds—in particular any changes in the number of seasonal migrants—is one of the key distinguishing attributes of its biodiversity when threatened by anthropogenic factors [15]. By formally documenting this information, researchers may help promote the potential for ecotourism, as well as compensation for farmers [16].

### Objectives

Contested areas such as GWCR are recognized as “war zone refugia” [3], but fauna within are not well-documented. Our objectives were to seasonally document the residential status, relative abundance, richness, evenness and feeding guilds of waterbirds in the GWCR over a full year. This information represents an essential step in a comprehensive plan to document the wildlife in this protected region, and to obtain baseline data for longitudinal comparisons of secure sanctuaries.

## Main text

### Methods

Gharana 32°32′28″N; 74°41′27″E; 281 m above sea level, located on the Indo-Pakistan border in the south-western part of Jammu and Kashmir, is composed of a rain-fed swamp with a bottom surface of loamy clay with decaying vegetation. It is in the subtropical climatic zone where summer temperatures reach 46 °C maximum and winter minima decrease to 2 °C. Annual rainfall is ~ 1331 mm, with the majority of precipitation occurring when the south-western monsoon winds arrive (July–September). Vegetation includes *Eicchornia* spp., *Hydrilla* spp. [17] and the common reed (*Typha* spp.). Due to local development, there is also surface runoff from agricultural fields [18].

The agricultural fields adjacent to Gharana village also provides both suitable habitat, and concomitant threats, for a diverse group of bird taxa [19]. These characteristics make this protected area both accessible and economically important. This wetland is also located in a state known for outdoor activities and adventure (Jammu and Kashmir), and is internationally renowned for birdwatching and mountaineering [16].

#### Data collection

Our methods have been previously reported in the overarching project [19], except herein we report seasonal fluctuations limited to waterbirds, as the data was far too prodigious to include in one report. Twenty-four surveys were conducted from July 2012 to June 2013, covering all seasons; summer (April–June), monsoon (July–Sept), autumn (Oct–Novem) and winter (Dec–March). These surveys strictly followed well-established methods for line transects and point count methods in [20] (e.g., using widely-spaced, randomly-elected transects, with attention to avoiding bias from effort, walking speed, or weather conditions; birds flying overhead were counted separately as they cannot be used in density estimation). Counts were performed twice per month at all sites by a team of ten individuals in the early morning (07:00–10:00) during the time of highest bird activity [21] and lowest human disturbance. Observer effects were minimal because these animals have habituated to humans through agricultural and military actions. Experts > 200 h of wetland bird identification and post-doctoral training were consulted throughout the period.

All waterbird species were classified as common/rare, and also resident/migratory status of the birds as per [22]. For instance, VC = very common species encountered during (80% of all surveys); C = common species encountered frequently (50–70%) and R = rare species which are encountered less frequently (10–20%). Likewise, if a particular species was documented between December and March, then it was considered as a winter visitor. Whereas, presence between April and June was documented as a summer visitation. If we documented a waterbird was documented throughout a year in and around GWCR, then it was considered as a resident. Feeding guilds were identified from the literature, rather than what birds were seen feeding on at the time. Nikon Monarch 10 × 42 binoculars were used during surveys for taking observations and on-the-spot identification. Photographs and/or video were used to validate any unidentified species. The checklist was prepared using the standardized common and scientific names assigned in [23]. All data collected were observational and did not involve any manipulation or alteration of any animals, plants or humans.

#### Statistics

Univariate analysis of variance (ANOVA) was used to examine the relationships between season and between number of individuals and the number of species. Tukey’s post hoc test was used to test pair-wise comparisons between seasons. Univariate ANOVA was also used to examine the relationship between season and each of four indices (Shannon–Wiener, Simpson’s Diversity, Equitability J and the Margalef index). Cross tabulations with Pearson’s Chi Square tests were performed between feeding guild and abundance, feeding guild and residence status, and between abundance and residence status. We used linear regressions to assess the relationship individuals and temperature and between species and temperature. Statistical significance (alpha) was set at *P* ≤ 0.05 and descriptive and inferential analyses were conducted using Minitab V. 17 (State College, PA).

### Results

We documented 61 waterbird species from 11 families of 6 orders over 1 year (Table 1); 28 species were rare. The majority were from three families, the Anatidae (Anseriformes), Phalacrocoracida (Pelicaniformes), and Rallidae (Gruiformes). We found the most waterbird species in March (39), and fewest in June (16). The largest population (~ 9701 individuals) was also recorded in March while the lowest population size (130 individuals) was found in May. Order Anseriformes contributed the most species (19). During March, the Bar-headed Goose (*Anser indicus*) constituted 62% (6000 individuals) while the Ruddy Shelduck (*Tadorna ferruginea*) accounted for 15% (1500) of the total population (9701) count.

**Table 1:** Inventory of waterbirds of the Gharana Wetland Conservation Reserve recorded from July 2012 to June 2013

Sp.	Order	Family	Common name	Scientific name	Residential status	Abundance	Feeding
1	Podicipediformes	Podicipedidae	Little grebe	*Tachybaptus ruficollis*	R	VC	C
2	Pelecaniformes	Phalacrocoracidae	Great cormorant	*Phalacrocorax carbo*	WV	VC	C
3			Little cormorant	*P. niger*	WV	VC	C
4	Ciconiiformes	Ardeidae	Yellow bittern	*Ixobrychus sinensis*	WV	R	C
5			Black-crowned night-heron	*Nycticorax nycticorax*	WV	C	C
6			Indian pond heron	*Ardeola grayii*	R	VC	C
7			Cattle egret	*Bubulcus ibis*	R	VC	C
8			Little egret	*Egretta garzetta*	R	VC	C
9			Intermediate egret	*E. intermedia*	R	C	C
10			Great egret	*E. alba*	WV	C	C
11			Purple heron	*Ardea purpurea*	R	VC	C
12			Grey heron	*A. cinerea*	R	VC	C
13		Ciconiidae	Painted stork	*Mycteria leucocephala*	WV	R	C
14			Black stork	*Ciconia nigra*	WV	R	C
15			Wooly-necked stork	*C. episcopus*	WV	R	C
16			Black-necked stork	*Ephippiorhynchus asiaticus*	WV	R	C
17		Threskiornithidae	Black-headed ibis	*Threskiornis melanocephalus*	WV	R	C
18			Black ibis	*Pseudibis papillosa*	WV	R	C
19			Glossy ibis	*Plegadis falcinellus*	WV	R	C
20			Eurasian spoonbill	*Platalea leucorodia*	WV	R	C
21	Anseriformes	Anatidae	Lesser whistling duck	*Dendrocygna javanica*	WV	VC	H
22			Greylag goose	*Anser anser*	WV	R	H
23			Greater white-fronted goose	*Anser albifrons*	WV	R	H
24			Bar-headed goose	*A. indicus*	WV	C	H
25			Ruddy shelduck	*Tadorna ferruginea*	WV	R	H
26			Knob-billed duck	*Sarkidiornis melanotos*	WV	R	H
27			Eurasian wigeon	*Anas penelope*	WV	C	H
28			Gadwall	*A. strepera*	WV	VC	H
29			Eurasian teal	*A. crecca*	WV	VC	H
30			Mallard	*A. platyrhynchos*	WV	R	H
31			Indian spot-billed duck	*A. poecilorhyncha*	WV	R	H
32			Northern pintail	*A. acuta*	WV	C	H
33			Garganey	*A. querquedula*	WV	R	H
34			Northern shoveler	*A. clypeata*	WV	VC	H
35			Red-crested pochard	*Netta rufina*	WV	R	H
36			Common pochard	*Aythya ferina*	WV	C	H
37			Ferruginous duck	*A. nyroca*	WV	R	H
38			Tufted duck	*A. fuligula*	WV	R	H
39	Gruiformes	Rallidae	Water rail	*Rallus aquaticus*	WV	C	O
40			White-breasted waterhen	*Amaurornis phoenicurus*	R	VC	O
41			Common moorhen	*Gallinula chloropus*	R	VC	O
42			Purple swamphen	*Porphyrio porphyrio*	R	VC	O
43			Eurasian coot	*Fulica atra*	WV	C	O
44		Gruidae	Common crane	*Grus grus*	WV	R	O
45	Charadriiformes	Jacanidae	Pheasant-tailed jacana	*Hydrophasianus chirurgus*	SW	C	O
46		Charadriidae	Red-wattled lapwing	*Vanellus indicus*	R	VC	O
47			White-tailed lapwing	*V. leucurus*	WV		
48			Little ringed plover	*Charadrius dubius*	R	R	O
49		Scolopacidae	Common snipe	*Gallinago gallinago*	WV	R	I
50			Common sandpiper	*Actitis hypoleucos*	WV	C	I
51			Green sandpiper	*Tringa ochropus*	WV	R	I
52			Common greenshank	*T. nebularia*	R		
53			Curlew sandpiper	*Calidris ferruginea*	V	R	I
54			Little stint	*C. minuta*	V	R	I
55			Ruff	*Philomachus pugnax*	WV	VC	I
56		Recurvirostridae	Black-winged stilt	*Himantopus himantopus*	WV	C	I
57		Glareolidae	Oriental pratincole	*Glareola maldivarum*	V	R	I
58			Little pratincole	*G. lactea*	R	C	I
59		Laridae	River tern	*Sterna aurantia*	SW	C	C
60			Common tern	*S. hirundo*	V	R	C
61			White-winged tern	*Chlidonias leucopterus*	V	R	C

Feeding guilds: *I* insectivorous, *O* omnivorous, *C* carnivorous, *H* herbivorous; Residential status: *WV* winter visitors, *R* resident, *V* vagrant, *SV* summer visitors; Abundance: *C* common, *VC* very common, *R* rare

The number of waterbird individuals (ANOVA *F*_3,731_ = 3.82; *P *< 0.01) and species (ANOVA *F*_3,11_ = 5.81; *P* < 0.05) varied by season (Table 2). Tukey’s post hoc test showed that the number of individuals and species in the Winter differed to all three seasons, and that Autumn, Monsoon, and Summer seasons were not different to one another. Among the 4 indices, the Shannon–Wiener (ANOVA *F*_3,11_ = 25.2; *P* < 0.001), Simpson’s Diversity ANOVA *F*_3,11_ = 18.5; *P *< 0.001, and Equitability J (ANOVA *F*_3,11_ = 18.6; *P* < 0.001) were each significantly different across seasons. In all cases winter was different to the other three seasons. The Margalef index (ANOVA *F*_3,11_ = 0.75; *P* > 0.5) did not vary significantly by season.

**Table 2:** Seasonal variations in species diversity, dominance and evenness of waterbirds by month/season identified in the Gharana Wetland Conservation Reserve from July 2012 to June 2013

Season	Month	Individuals	Species	Shannon–Wiener	Simpson’s 1-D	Margalef	Equitability J
Monsoon	July	142	17	2.67	0.92	3.23	0.94
	August	235	28	3.03	0.94	4.95	0.91
	September	276	28	3.13	0.95	4.80	0.94
Autumn	October	483	32	3.25	0.96	5.02	0.94
	November	841	31	2.96	0.92	4.46	0.86
Winter	December	2091	32	1.60	0.54	4.06	0.46
	January	2347	36	1.31	0.44	4.51	0.36
	February	5028	37	1.31	0.50	4.22	0.36
	March	9701	39	1.52	0.59	4.14	0.41
Summer	April	513	29	1.92	0.65	4.49	0.57
	May	133	21	2.70	0.90	4.09	0.89
	June	130	16	2.60	0.91	3.08	0.94

Index (year)	Mean	± SE	Min	Q1	Q3	Max	
Shannon–Weaver	2.33	0.03	1.31	1.54	3.01	3.25	
Simpson	0.77	0.01	0.44	0.55	0.94	0.96	
Margalef	4.25	0.02	3.08	4.06	4.73	5.02	
Equitability J	0.72	0.01	0.36	0.43	0.94	0.94	

Among the 61 species, 15 were common (25%), 28 were rare (46%), and 18 very common 29% (Table 1). Within feeding guilds, among the 18 species of carnivores, 8 species were ‘very common’ (44%), however cross tabulations (Table 3) showed no association between feeding guild and abundance (χ^2^= 4.4; P > 0.5). There was an association between feeding guild and residence status (χ^2^= 21.9; P < 0.001). Winter visitors were more likely to be herbivores (46%), whereas there were no resident herbivores. There was also an association between residence status and abundance (χ^2^ = 21.9; P < 0.001) (Table 3). Rare waterbirds were most likely to be winter visitors (54%) than were common (26%) or very common birds (21%). Among the 14 species of residents, 10 were very common (71%) (Table 3). There was a strong negative correlation between temperature and number of species *S* = 4.84; *R*^2^= 61.8%; *F* = 16.15; *P* < 0.005. No correlation was found between temperature and number of individual waterbirds; *S* = 2803.18; *R*^2^= 13.3%; *F *= 1.53; *P* > 0.2.

**Table 3:** Frequency distribution and relative percentages of each feeding guild of waterbirds in relation to abundance and residential status (above)

	Feeding guild				χ^2^	*P*
	Carnivore	Herbivore	Insectivore	Omnivore		
Abundance						
Common	4 (26.7%)	4 (26.7%)	4 (26.7%)	3 (20%)	4.40	> 0.5
Rare	10 (35.7%)	10 (35.7%)	5 (17.8%)	3 (10.7%)		
Very common	8 (44.4%)	5 (27.7%)	1 (5.5%)	4 (22.2%)		
Resident status						
Resident	7 (50%)	0 (0%)	2 (14.2%)	5 (35.7%)	21.9	< 0.001
Summer visitor	1 (33.3%)	1 (33.3%)	0 (0%)	1 (33.3%)		
Vagrants	2 (40%)	0 (0%)	3 (60%)	0 (0%)		
Winter visitor	12 (30.7%)	18 (46.1%)	5 (12.8%)	4 (10.2%)		

	Abundance				*P*	
	Common	Rare	Very common	χ^2^		
Resident status						
Resident	3 (21.4%)	1 (7.1%)	10 (71.4%)	23.6	< 0.001	
Summer visitor	2 (66.7%)	1 (33.3%)	0 (0%)			
Vagrants	0 (0%)	5 (100%)	0 (0%)			
Winter visitor	10 (25.6%)	21 (53.8%)	8 (20.5%)			

Frequency distribution and relative percentages of each resident status according to abundance (below)

### Discussion

Protected fauna inhabiting “war zone refugia” have not been well documented [3], despite their presence and vulnerability in geographically-contested areas worldwide [3]. Here, we have provided a template for obtaining baseline data for waterbirds living in such areas. In doing so, we have provided the first documentation of the seasonal status, relative abundance, species richness, evenness and dominance of waterbirds under-duress over 1 year. We have identified 61 species from 11 families of 6 orders; two-thirds of all species (40) were visitors, and almost half (28) were rare. Waterbirds present during the stopover period (March high-altitude return migration) contributed more to the four indices than over-wintering birds (late Autumn upsurge).

Like most wetlands, this reserve supports birds of a diverse array of ecological niches and therefore, varied diets. The majority of resident species were carnivores, likely owing to the wide availability of year-round access to invertebrate fauna. Most of the carnivores were very common, whereas there were no resident herbivores. However, migrants were more likely to be herbivores, partly explaining many locals’ frustration with the loss of crops. Furthermore, most of the herbivores visited during the winter when birds predate the young and highly palatable shoots of wheat and therefore inflict maximum damage to crops. This dynamic demonstrates that either the presence and absence of military activity in such areas can result in direct duress from shelling, or indirectly result in agricultural duress when farmers move forward once the shelling periods cease.

Continued documentation of the avian fauna and their availability of resources is necessary to aid in the promotion of the wetland for improved conservation. While important for baseline data and continual monitoring, this information may also be utilized to quantify numbers to inspire ecotourism and similar approaches to enhance the livelihood of resident farmers and provide alternatives to farming for income. At the global scale, conservationists should pay special attention to document avifauna in contested regions, or militarized borders, while not prematurely assuming that species in protected sanctuaries are safe from duress.

## Limitations

Our data are descriptive, consider ad hoc hypotheses, and do not include comparative data from other wildlife refuges or sanctuaries around the globe. Further, we do not report quantifiable measures of shelling (e.g., number of explosions, amplitude of noise generated, lethality, damage to young, or variation by season). However, we hope our initial communication encourages others to analyse and report the well-being of fauna in both secure and vulnerable sanctuaries in the presence and absence of militarization.

## Abbreviations

GWCR: Gharana Wetland Conservation Reserve
IBA: Important Bird Area

## References

[1] Gese EM, Rongstad OJ, Mytton WR (1989). Changes in coyote movements due to military activity. J Wildl Manag.

[2] Krausman PR, Harris LK, Blasch CL, Koenen KK, Francine J (2004). Effects of military operations on behavior and hearing of endangered *Sonoran pronghorn*. Wildl Monogr.

[3] Dudley JP, Ginsberg JR, Plumptre AJ, Hart JA, Campos LC (2002). Effects of war and civil strife on wildlife and wildlife habitats. Conserv Biol.

[4] Wright RG (1999). Wildlife management in the national parks: questions in search of answers. Ecol Appl.

[5] Zakaria M, Rajpar MN (2010). Bird species composition and feeding guilds based on point count and mist netting methods at the Paya Indah Wetland Reserve, Peninsular Malaysia. Trop Life Sci Res.

[6] Veen J, Yurlov A, Delany S, Mihantiev A, Selivanova M, Boere G (2005). An atlas of movements of Southwest Siberian waterbirds.

[7] Schuyt K, Brander L (2004). The economic values of the world’s wetlands, living waters.

[8] Zafar-ul-Islam M, Rahmani AR (2004). Important bird areas in India: priority sites for conservation.

[9] Mahapatra DA: Positioning the People in the Contested Borders of Kashmir. Centre for international border studies research working paper. 2011. p. 21.

[10] Saving Gharana Sanctuary. Jammu Kashmir Latest News|Tourism|Breaking News J&K. http://www.dailyexcelsior.com/saving-gharana-sanctuary/. Accessed 15 Dec 2017.

[11] Loucks C, Mascia MB, Maxwell A, Huy K, Duong K, Chea N, Long B, Cox N, Seng T (2009). Wildlife decline in Cambodia, 1953–2005: exploring the legacy of armed conflict. Conserv Lett.

[12] Nyhus PJ, Osofsky SA, Ferraro P, Madden F, Fischer H (2005). Bearing the costs of human–wildlife conflict: the challenges of compensation schemes. Conserv Biol Ser Camb.

[13] Rahmani AR, Kalra M, Khan NI (2012). Threatened birds of India: their conservation requirements.

[14] Aung M, Swe KK, Oo T, Moe KK, Leimgruber P, Allendorf T, Duncan C, Wemmer C (2004). The environmental history of Chatthin Wildlife Sanctuary, a protected area in Myanmar (Burma). J Environ Manag.

[15] Burger J (1985). Habitat selection in temperate marsh-nesting birds.

[16] Sharma A (2014). Ecotourism in J&K: vehicle to sustainable development. Rev Res J.

[17] Tara J, Kour R, Sharma S (2011). A record of aquatic Hemiptera of Gharana Wetland, Jammu. Bioscan.

[18] Pandotra A, Sahi D (2014). Avifaunal assemblages in suburban habitats of Jammu, J&K, India. Res J Environ Sci.

[19] Jamwal PS, Chandan P, Rattan R, Anand A, Kannan PM, Parsons MH (2017). Survey of Avifauna of the Gharana Wetland Reserve: implications for conservation in a semi-arid agricultural setting on the Indo-Pakistan border. BMC Zool.

[20] Bibby CJ (2000). Bird census techniques.

[21] Buckland ST, Anderson DR, Burnham KP, Laake JL, Borchers DL, Thomas L (2001). Introduction to distance sampling estimating abundance of biological populations.

[22] Saikia P, Saikia M (2000). Diversity of bird fauna in NE India. J Assam Sci Soc.

[23] Kazmierczak K, Perlo BV (2000). Field guide to the birds of the Indian Subcontinent.

